# Ce-Containing MgAl-Layered Double Hydroxide-Graphene Oxide Hybrid Materials as Multifunctional Catalysts for Organic Transformations

**DOI:** 10.3390/ma14237457

**Published:** 2021-12-04

**Authors:** Alexandra-Elisabeta Stamate, Octavian Dumitru Pavel, Rodica Zăvoianu, Ioana Brezeştean, Alexandra Ciorȋță, Ruxandra Bȋrjega, Katja Neubauer, Angela Koeckritz, Ioan-Cezar Marcu

**Affiliations:** 1 Department of Organic Chemistry, Biochemistry & Catalysis, Faculty of Chemistry, University of Bucharest, 4-12, Blv. Regina Elisabeta, 030018 Bucharest, Romania; alexandra-elisabeta.stamate@drd.unibuc.ro (A.-E.S.); octavian.pavel@chimie.unibuc.ro (O.D.P.); 2 Research Center for Catalysts & Catalytic Processes, Faculty of Chemistry, University of Bucharest, 4-12, Blv. Regina Elisabeta, 030018 Bucharest, Romania; 3 National Institute for Research and Development of Isotopic and Molecular Technologies, 67-103 Donat, 400293 Cluj-Napoca, Romania; ioana.brezestean@itim-cj.ro (I.B.); al.ciorita@yahoo.com (A.C.); 4 Biomolecular Physics Department, Faculty of Physics, Babes-Bolyai University, 1, Kogălniceanu Str., 400084 Cluj-Napoca, Romania; 5 Electron Microscopy Centre, Faculty of Biology and Geology, Babes-Bolyai University, 44, Republicii Str., 400015 Cluj-Napoca, Romania; 6 National Institute for Lasers, Plasma and Radiation Physics, 409 Atomistilor Street, P.O. Box MG-16, 077125 Măgurele, Romania; ruxandra.birjega@inflpr.ro; 7 Leibniz Institute for Catalysis (LIKAT Rostock), Albert-Einstein-Straße 29a, 18059 Rostock, Germany; katja.neubauer@catalysis.de (K.N.); angela.koeckritz@catalysis.de (A.K.)

**Keywords:** hybrid materials, layered double hydroxides, graphene oxide, Knoevenagel condensation, tandem reactions, benzyl alcohol oxidation, one-pot cascade reactions

## Abstract

The combination of layered double hydroxides (LDH) with graphene oxide (GO) enables the formation of nanohybrids with improved properties. This work focuses on the structural and catalytic properties of Ce-containing MgAl LDH-GO composites bearing different concentrations of GO in the range of 5–25 wt.%. The synthesis of the composites was performed by co-precipitating the LDH phase in the presence of GO, while their characterization was performed using XRF, XRD, DRIFT, Raman, SEM, nitrogen adsorption-desorption, and acidity-basicity measurements. The LDH-GO composites, showing redox, basic, and acid catalytic functions, were tested in two different types of organic transformations: (i) Knoevenagel condensation and (ii) one-pot cascade oxidation-Knoevenagel condensation. (i) The cinnamic acid was synthesized by the Knoevenagel condensation of benzaldehyde with diethylmalonate. The composites showed catalytic performances in strong contrast to neat LDH or GO, suggesting a synergistic interaction between the two components. During Knoevenagel condensation, the catalytic activity increased with the GO content in the hybrids up to 15 wt.% and decreased afterwards. (ii) 2-Benzoyl-3-phenylacrylonitrile was synthesized by the aerobic oxidation of benzyl alcohol followed by the Knoevenagel condensation with benzoyl acetonitrile using three different non-polar solvents, i.e., toluene, benzene, and mesitylene. The conversion of benzyl alcohol was higher for the hybrid materials compared to the individual components but decreased with the increase of the graphene oxide concentration.

## 1. Introduction

Developing improved strategies for the manufacture of two-dimensional (2D) nano-hybrid multifunctional materials has attracted a lot of interest in advanced research and technology. The combination of various nanomaterials having different physical and chemical properties can lead to improved nanocomposites possessing the qualities of the parent building blocks [1]. Recently, hybrid materials have gained a lot of attention due to their usefulness in various applications, from industry to water treatments and catalysis [1,2]. Graphene oxide and layered double hydroxides are among the various compounds that can be combined [2].

Layered double hydroxides (LDH), also called hydrotalcite (HT)-like compounds, are anionic clays having the general formula [M^II^_1-x_M^III^_x_(OH)_2_]^x+^[A^n−^]_x/n_·zH_2_O, where M^II^ is a bivalent metal cation and M^III^ is a trivalent metal cation, A^n−^ is a charge-compensating anion which may be either inorganic or organic, and *x* has a value between 0.2 and 0.4. LDH solids are formed of positively charged brucite-type layers containing the cations hexacoordinated with hydroxyl groups [3]. Between two brucite-type layers, there is a space called the interlayer region containing the compensation anions and water molecules. The combination of layered double hydroxides (LDH) with components such as graphene oxide (GO), a 2D honeycomb material with oxidizing properties [4,5], can lead to the formation of nanohybrids with improved properties that can be valorized in many applications, including catalysis [1,2]. Despite its advantageous properties such as increased optical, electronic, and thermal stability [4,6], graphene oxide tends to agglomerate and restack [1,4]. Thus, to overcome this problem, it is necessary to embed GO into other materials, such as layered double hydroxides. Graphene oxide and LDH present different chemical and physical properties which complement each other up to a certain point offering hybrid materials that have layered structure, large surface area, multiple functional groups coming from GO, and the anionic exchangeability of the hydrotalcites [6].

Although tremendous progress has been made, numerous hurdles still need to be overcome before hybrid materials containing GO and LDH can be used to their full potential. Although the present synthetic approaches seem promising, the materials must be generated on an industrial level to achieve large-scale production.

Carbon–carbon bond formation has been considered as the fundamental key of the entire organic chemistry [7]. One of the most efficient ways to obtain the desired covalent bond has proven to be aldol condensation, which is generally catalyzed by bases, acids, or catalysts presenting both basic and acidic sites [8]. The base-catalyzed aldol condensation, known as the Knoevenagel condensation, has been widely exploited to produce various specialty chemicals, intermediates with biological applications, or valuable products such as calcium antagonist drugs, and other pharmaceuticals, polymers, coumarin derivatives, and even for the beauty industry (cosmetics, perfumes) [7].

In the Knoevenagel condensation reaction, an aldehyde or a ketone condenses with active methylene-containing compounds (acyclic 1,3-dicarbonyls and derivatives such as malonates, acetoacetates, acetonitriles, acetylacetone, malononitrile, or cyclic CH-acidic compounds such as 1,3-cyclohexanediones, Meldrum’s acid, barbituric acids, oxazepanediones, 4-hydroxycoumarin, etc.) usually in the presence of alkali metal hydroxides, organic bases (primary, secondary, and tertiary amines) or their corresponding ammonium salts, urea, pyridine, and different Lewis basic/acidic materials [8,9,10,11,12]. However, the condensation process, conventionally performed under homogenous conditions, has some disadvantages associated to the inability of the homogeneous catalysts to be recovered and recycled or to the excess of the solvent used during the reaction, which may generate increased volumes of toxic waste affecting the environment [8,10]. To overcome these drawbacks, a large variety of solid catalysts has been exploited over the past years such as polystyrene-supported poly(amidoamine) dendrimers [13], polystyrene immobilized DABCO [14], various amine/diamine-functionalized materials [15,16,17,18], chitosan hydrogel [19], acrylic resin-immobilized lipase [20], zeolites [21,22,23], metal-organic frameworks [24], diethylenetriamine-functionalized graphene oxide with Fe_3_O_4_ nanoparticles [25], and many others. Nevertheless, these catalytic systems still present some inconveniences, such as the leaching of the basic fragment of the catalyst, the active site blocking, and the redundant use of organic solvents and additives [12]. Considering these limitations, the development of an environmentally friendly, efficient, and selective heterogeneous catalytic system that presents a high activity for the Knoevenagel reaction under mild conditions continues to be a challenging research domain. 

One-pot cascade reactions, also called tandem reactions, represent an ingenious solution that can drastically simplify and improve intricate synthetic pathways, also reducing the production of wastes and energy consumption [26]. One-pot cascade reactions must be carefully planned to ensure that the correct sequence of reactions is followed in the correct order to get the target product. Designing a suitable multifunctional catalyst having various, spatially isolated active sites is essential for a successful tandem reaction. It is critical to select an appropriate material for the development of a multi-functional catalyst that has some catalytic activity of its own and that also possesses the ability to integrate some extra catalytic features. To date, a wide variety of catalytic systems have been proposed for cascade reactions, however, many of these have been based on homogeneous catalysts [26,27]. Compared to homogeneous catalysts, the heterogeneous ones present important advantages, such as easy recovery and recyclability, less contamination of the reaction medium, and increased stability [26,27]. The heterogeneous solids that have been reported in the literature as catalysts for the alcohol oxidation/Knoevenagel condensation cascade reactions, such as Pd-nanoparticle-supported Nano-ZSM-5 [28], Pd/LS-AT-OH@catalyst [29], Pd0@UiO-68-AP [30], porphyrin-based microporous organic polymer Fe-POP-1 [31], polyoxometalate-intercalated LDH [26], Pd1-Au1/LDH [32], NiGa LDHs [33], and copper complexes [34], presented some drawbacks which referred to high costs of the synthesis, high-temperature regimes, and the use of basic additives during the tandem reactions. 

Considering the already published data, a potential class of catalysts that exhibits the necessary requirements to obtain promising results not only in Knoevenagel condensations but also in oxidation/Knoevenagel condensation cascade reactions is represented by the layered double hydroxides. Developing a highly efficient catalytic system for the aerobic oxidation of alcohols to the corresponding carbonyl compounds without any additives is particularly attractive in synthetic and industrial organic chemistry [26]. Recently, layered double hydroxides modified/doped with rare-earth elements have received increasing attention due to their enhanced basicity which led to improved catalytic performance [35]. However, the beneficial catalytic, electric, and magnetic properties generated by the insertion of rare-earth metals cannot be exploited to their full potential due to the challenging incorporation of large rare-earth cations into the brucite structure, as in the case of cerium ions which possess a higher ionic radius compared to the main cations involved in the construction of the brucite-like layers [36]. Moreover, according to the literature, the synthesis of single-phase Ce-modified LDHs is quite difficult since cerium (III) is easily oxidized in aqueous solutions [36]. Therefore, most of the samples contain supplementary phases such as CeCO_3_OH or CeO_2_ [36]. 

**Scheme 1:**
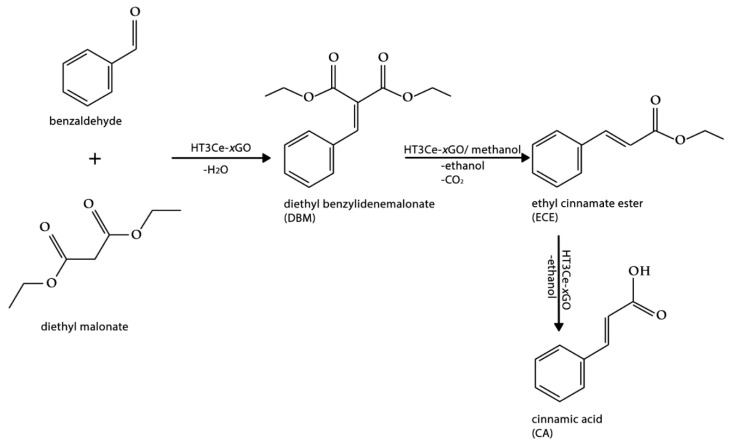
Reaction route of Knoevenagel condensation.

**Scheme 2:**
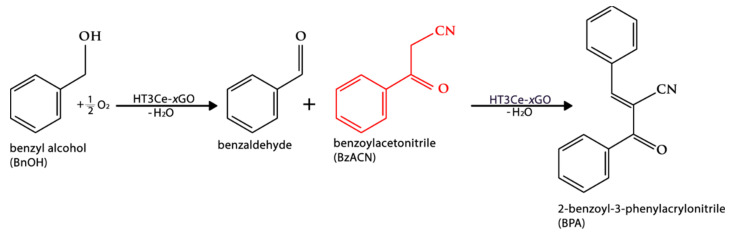
Reaction route for one-pot cascade oxidation–Knoevenagel condensation reactions.

The current study focuses on the synthesis and structural and catalytic properties of Ce-containing MgAl LDH-GO composites bearing different concentrations of GO in the range of 5–25 wt.% abbreviated as HT3Ce-xGO where *x* stands for the concentration of GO (*x* = 5, 10, 15, 15, 20, 25 wt.%). The solids were tested as catalysts in two different types of organo-chemical transformations: (i) Knoevenagel condensation reaction (Scheme 1) and (ii) one-pot cascade oxidation-Knoevenagel condensation (Scheme 2). Notably, by reviewing the literature, only two articles containing LDH-GO hybrids, with the layered double hydroxide modified with a lanthanide (La [37], and Gd [38], respectively) stood out. Moreover, these hybrids had no catalytic purpose, being only used as flame-retardant [37] and nano-carriers for magnetic resonance imaging and drug delivery [38]. 

## 2. Materials and Methods

### 2.1. The Synthesis of Graphene Oxide (GO)

The method applied for the preparation of graphene oxide is based on the technique developed by Hummers in 1952 [39]. Thus, graphite powder (325 mesh, from Aldrich, Schnelldorf, Germany), sodium nitrate (NaNO_3_) and potassium permanganate (KMnO_4_, chemical purity, from Merck, Darmstadt, Germany), sulfuric acid (H_2_SO_4_, 98%, from Merck, Darmstadt, Germany), hydrochloric acid (HCl, 37%, from Merck), and hydrogen peroxide (H_2_O_2_, 30%, from Chimreactiv, Bucharest, Romania) were utilized. In brief, 23 mL concentrated sulfuric acid and 1 g of graphite were mixed in an Erlenmeyer flask maintained in an ice bath at 0 °C. Then, 0.5 g of sodium nitrate and 3.0 g of potassium permanganate were slowly added to the formed solution which was kept under continuous stirring for 30 min.

Next, the conical flask was maintained for another 30 min in the thermostat at 30–40 °C. As the reaction progressed, the mixture gradually thickened turning into a green paste. Further, the paste was diluted with 46 mL of distilled water causing a strong exothermic effect accompanied by bubbling, and the resulting mixture was stirred for one hour at 90 °C. Moreover, to remove the residual permanganate, the whole solution was mixed with 71 mL of warm distilled water and 5 mL H_2_O_2_ and kept for one hour under stirring. For the removal of the remaining metal ions, 125 mL HCl 0.1 N was added, and the solution was stirred for another hour. The resulting mixture was centrifuged and washed with warm distilled water until the conductivity was below 100 μS/cm.

The concentration of GO in the suspension was determined using the gravimetric method, by weighing 3 liquid samples of 100 mL in Petri dishes before and after the evaporation of water under vacuum at 60 °C for 24 h. The amount of solid recovered from the 3 samples was 0.4001, 0.4005, and 0.3998 g, which gave an average value of the GO concentration in the suspension of ca. 4 g/L.

### 2.2. The Preparation of The LDH-GO Composites

The Ce-containing MgAl LDH-GO composites bearing different concentrations of graphene oxide (GO) in the range of 5–25 wt.% labeled HT3Ce-xGO, where *x* stands for the concentration of GO (*x* = 5, 10, 15, 20, 25 wt.%), were synthesized by co-precipitation of the LDH phase in the presence of GO suspension at pH of 10. 

The preparation steps of this method were identical for all the composites, the only difference in their synthesis were the amounts of precursor salts used, which varied depending on the concentration of graphene oxide aimed to be included in the resulting solid. To obtain the solid LDH-GO compounds, two aqueous solutions were necessary: solution A containing 1.5 M of cations (Mg^2+^, Al^3+^, and Ce^3+^, with the molar ratio Mg^2+^/Al^3+^/Ce^3+^ of 3/0.75/0.25) dissolved in the appropriate amount of GO suspension, and B containing NaOH and Na_2_CO_3_ (1 M concentration of Na^+^ ions, with a molar ratio NaOH/Na_2_CO_3_ of 2.5/1).

The two solutions, A and B, were mixed in a round-bottomed flask under continuous stirring, then kept for aging for 18 h. The washing of the product was carried out with distilled water until the conductivity was below 100 μS/cm. Drying was carried out in an oven at a temperature of 90 °C in an air atmosphere for 24 h. The resulting LDH-GO solids containing 5, 10, 15, 20, 25 wt.% GO were abbreviated as follows: HT3Ce-5GO, HT3Ce-10GO, HT3Ce-15GO, HT3Ce-20GO, and HT3Ce-25GO, respectively.

A Ce-containing hydrotalcite unmodified with graphene oxide was also prepared by co-precipitation under the same above-described conditions, but in this case, solution A was obtained with distilled water instead of GO suspension. The resulting compound was abbreviated as HT3Ce.

### 2.3. Materials Characterization

X-ray fluorescence data were collected using a Panalytical Epsilon 1 spectro-meter (from Panalytical, Almelo, Netherlands) operated with an Ag X-ray source. 

XRD powder patterns were recorded on a Panalytical X’Pert θ/2θ-diffractometer (from Panalytical, Almelo, Netherlands) equipped with Xcelerator detector using automatic divergence slits and Cu-Kα1/α2 radiation (40 kV, 40 mA; λ = 0.15406 nm, 0.154443 nm). Cu beta-radiation was excluded using a nickel filter foil. The measurements were performed at 0.32°/min. Samples were mounted on silicon zero background holders. Obtained intensities were converted from automatic to fixed divergence slits (0.25°) for further analysis. Peak positions and profiles were fitted with the Pseudo-Voigt function using the HighScore Plus software package (Panalytical, Almelo, Netherlands). Phase identification was done by using the PDF-2 database of the International Center of Diffraction Data (ICDD).

The total number of acid sites was determined by pyridine adsorption from the areas of the corresponding peaks in the DRIFT spectra recorded on JASCO FT/IR-4700 spectrometer (Tokyo, Japan). Based on the literature, the bands corresponding to pyridine adsorbed on Lewis acid sites appear in the ranges of 1435–1455 cm^−1^ and 1570–1615 cm^−1^, while those corresponding to pyridine adsorbed on Brønsted acid sites appear in the range of 1520–1555 cm^−1^ and at 1620–1630 cm^−1^ [40,41,42]. Samples of dried solids (0.05 g) were contacted with pyridine aliquots (0.2 μL each) and maintained under inert flow at 90 °C for the removal of physisorbed pyridine. The procedure was repeated until the weight of the sample after two consecutive additions of pyridine was constant (did not vary with more than 0.0001 g). Then, the DRIFT spectrum of the sample with adsorbed pyridine was recorded considering the DRIFT spectrum of the freshly dried solid as background. 

The total number of base sites of the compounds was determined by the irreversible adsorption of acrylic acid with pKa = 4.2, followed by the quantitative determination of the adsorbed acid using a UV–Vis spectrometer (JASCO V650, Tokyo, Japan). 

The DRIFT spectra of the solids synthesized were recorded with JASCO FT/IR-4700 spectrometer having a PIKE accessory for diffuse reflectance, they were collected in the spectral range of 4000–400 cm^−1^, with a scanning speed of 128 scans/min and a resolution of 4 cm^−1^ using KBr as background.

A high-resolution confocal Raman microscope (Renishaw system, from Renishaw Ltd. New Mills Wotton-under-Edge, Gloucestershire, UK) equipped with two laser lines (514 nm and 785 nm) and a Leica DM2500 microscope was utilized for recording the Raman spectra of the solid samples, in extended mode using the 514 nm laser line, monitoring the shifts in the Raman band position narrower than 0.5 cm^−1^, and measuring the Raman bands in the range of 100–3100 cm^−1^. 

All the samples were analyzed by scanning electron microscopy (SEM) using a Hitachi SU8230 (Hitachi, Tokyo, Japan) microscope at an acceleration voltage of 30 kV after the samples were covered with a 9-nm-thick layer of gold, using the Quorum Q150T ES turbomolecular pumped coater (Quorum Technologies, London, UK). The samples were coated with gold before analysis. To emphasize the interesting aspects, the secondary electrons signal was mixed with the backscattered electrons signal in equal ratios.

### 2.4. Catalytic Tests

The LDH-GO composites bearing different concentrations of GO in the range of 5–25 wt.% were tested as catalysts in two different types of organo-chemical transformations: (i) Knoevenagel condensation reaction (Scheme 1) and (ii) one-pot cascade oxidation-Knoevenagel condensation (Scheme 2).

All the Knoevenagel reactions were performed in a 100 mL stirred flask under reflux conditions at a temperature of 160 °C for two different reaction times, i.e., 5 h and 24 h. The catalytic activity of both GO and HT3Ce solids was also tested in the Knoevenagel condensation. 

In a typical experiment, at a molar ratio of 2:3, benzaldehyde (10 mmol, 1.02 mL) and diethyl malonate (15 mmol, 2.88 mL) were stirred and heated in a silicon oil bath. All reagents were purchased from Merck. In all reactions, the catalyst concentration was 1 wt.% in the reaction admixture, namely 0.346 g. After 5 and 24 h of reaction under reflux, respectively, the reactor was cooled to room temperature and 50 mL methanol was added to keep all the organic compounds in solution (heated again under reflux for 10 min). The catalysts were removed by filtration, while the organic phase was concentrated under vacuum and analyzed by mass spectrometer-coupled chromatography, using a GC/MS/MS Varian Saturn 2100 T (Varian, Palo, Alto, CA, USA) equipped with a CP-SIL 8 CB Low Bleed/MS column of 30 m length and 0.25 mm diameter. 

The same catalysts were investigated in one-pot cascade oxidation-Knoevenagel condensation reactions (Scheme 2). The tandem reactions were performed in a 100 mL Büchi miniclave low-pressure reactor (from Buchiglas (Büchi AG), Uster, Switzerland). The optimum temperature for both steps, oxidation and condensation, was 80 °C, and a further increase of temperature did not have any positive effect on the rate of the reaction. Regarding the reaction time, the ideal time for the oxidation step was 6 h, while the optimum for the condensation step was 19 h.

During the oxidation step, 1 mmol of benzyl alcohol (BnOH) was placed in the autoclave and mixed with 1 g of catalyst and 10 mL of solvent (toluene, benzene, or mesitylene). Then, the mixture was stirred under 1 atm of O_2_ and heated using a glycerin bath. After 6 h, the miniclave was opened and 1.2 mmol of benzoyl acetonitrile (BzACN) dissolved in 3 mL of solvent (toluene, benzene, or mesitylene) was added to the existing mixture to enable the condensation step and the formation of 2-benzoyl-3-phenylacrylonitrile (BPA). The miniclave was closed, purged with argon, and the combined mixture was stirred under 1 atm of argon for a further 19 h.

At the end of the reaction, the reactor was cooled to room temperature, the catalyst was removed by filtration and washed with acetonitrile, while the organic phase was analyzed by GC-MS using a SHIMADZU GCMS-QP2010 SE instrument (Shimadzu, Kyoto, Japan) equipped with an HP-5MS capillary column (30 m × 0.25 mm × 0.25 μm) from Agilent. Highly pure He (99.999%) was used as a carrier gas. 

## 3. Results and Discussions

### 3.1. Materials Characterization

The theoretical cationic composition of the Ce-containing hydrotalcite-type solid is described by the Mg/(Al + Ce) and Ce/Al atomic ratios which were fixed at 3 and 1/3, respectively. The obtained XRF results (Table 1) indicated that the Mg/(Al + Ce) ratio was lower than the theoretical value, in the range 2.63–2.92, while the Ce/Al ratio was close to the theoretical value, for all the materials, suggesting that the precipitation of magnesium was incomplete. At the same time, the cationic content of the materials continuously decreased with increasing the GO theoretical content (Figure S1) (Supplementary Materials), confirming the insertion of graphene oxide.

The results of the acidity and basicity measurements can also be seen in Table 1. It can be observed that the HT3Ce solid is mainly basic, while the GO is mainly acidic. The hybrid materials show, as expected, both basic and acidic sites, which enable them to act as bifunctional catalysts. The number of acidic sites passes through a maximum for the HT3Ce-15GO sample but remains lower than that corresponding to GO. On the other hand, the number of basic sites also passes through a maximum for HT3Ce-15GO but it is higher than that corresponding to the HT3Ce solid for all the hybrids. This suggests a synergistic interaction between the Ce-containing MgAl-LDH and the graphene oxide rather than a simple additive effect in the hybrid materials. Notably, among the hybrids, the solid HT3Ce-15GO not only contains the highest values of both total acidic and basic sites, but also shows the highest basic-to-acidic sites ratio. This is expected to be correlated with the catalytic results in the Knoevenagel condensation reactions.

**Figure 1:**
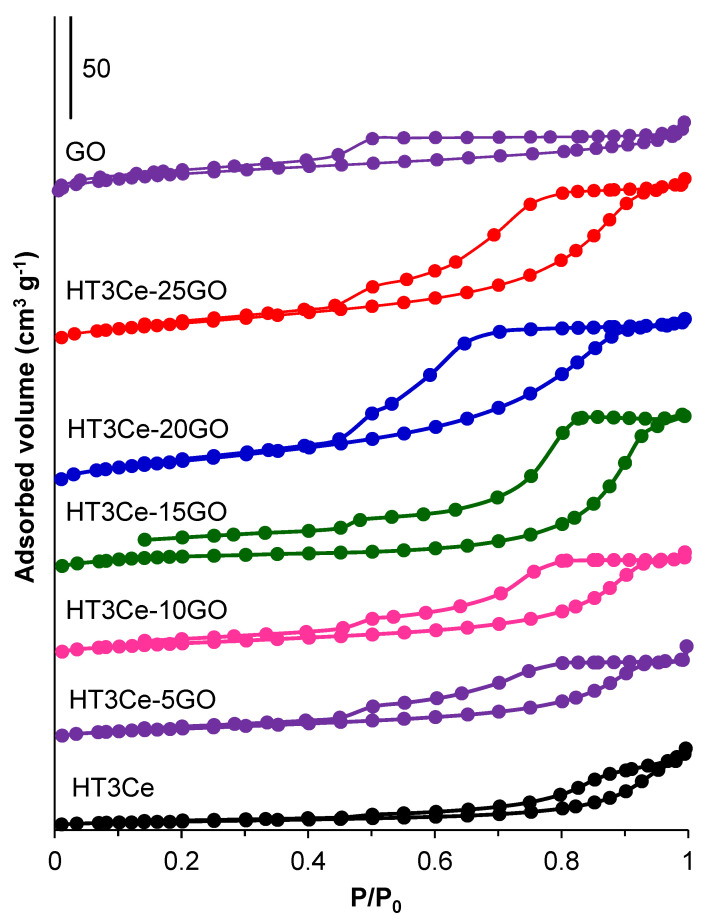
Nitrogen adsorption-desorption isotherms for HT3Ce LDH, GO, and HT3Ce-xGO samples.

The specific surface area, the pore volume, and the pore size of the HT3Ce LDH, GO and HT3Ce-xGO hybrids are tabulated in Table 2, and their corresponding adsorption-desorption isotherms and pore size distributions are shown in Figure 1 and Figure S2, respectively. All the samples reveal type IV isotherms according to the IUPAC classification, characteristic of mesoporous materials. However, the neat HT3Ce LDH shows a H3-type hysteresis loop attributed to aggregates of plate-like particles giving rise to slit-shaped pores, while the neat GO shows a H4-type hysteresis loop associated with narrow slit-like pores, including some microporosity [43]. At the same time, for the LDH-GO composites, the hysteresis loop moves towards H2b-type, which corresponds to more complex pore structures [44].

The data in Table 2 show that LDH has the lowest (15 m^2^/g), while GO has the highest (ca. 80 m^2^/g) surface area in this series, and, as expected, it increases by adding GO to the LDH and by increasing its content, suggesting alternative LDH-GO stacking interactions in the LDH-GO hybrids [45]. However, the surface area reaches a maximum of ca. 78 m^2^/g for the HT3Ce-20GO hybrid, then it decreases with ca. 10 m^2^/g for the HT3Ce-25GO hybrid obviously due to the GO restacking after a certain GO content in the hybrid. The pore volume of both LDH and GO samples are lower than that of the hybrids, and, for the latter, it increases by increasing the GO content.

The pore size distributions of the samples (Figure S2), obtained from the desorption branch of isotherms, indicate narrow and unimodal pore structures for GO (maximum at 38.9 Å), and broad and unimodal pore structures for the neat HT3Ce LDH and HT3Ce-5GO hybrid, with maxima at 126 and 186 Å, respectively. For the hybrid samples with higher GO content, narrower and bimodal pore size distributions are observed with maxima at 35–38 Å and 51–93 Å, respectively. The maxima of the large pores vary irrespective of the GO content in the LDH-GO composite materials.

**Figure 2:**
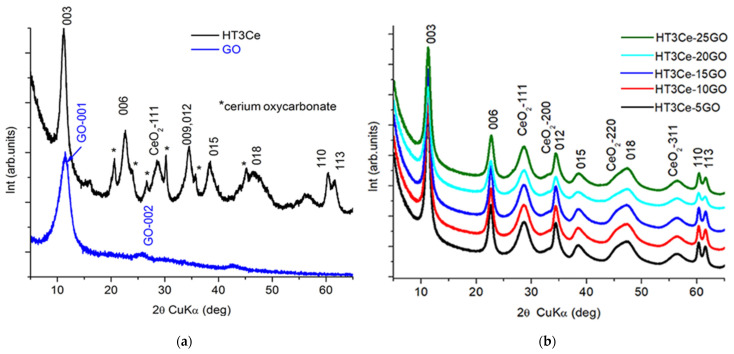
XRD patterns of: (**a**) HT3Ce and GO; (**b**) the composite samples.

The diffraction patterns of the solid samples, namely hydrotalcites obtained in the presence of different amounts of GO, pure GO, and HT3Ce are illustrated in Figure 2. The XRD pattern of the HT3Ce displays a mixture of characteristic reflections of a hexagonal LDH phase with rhombohedral 3R symmetry (ICDD card no. 054–1030), and of a cubic cerianite CeO_2_–phase (ICDD card no. 034–0394). They are Miller indexed consequently in Figure 2a. Additionally, diffraction lines assigned to cerium oxycarbonate (Ce_2_(CO_3_)_2_O·H_2_O, ICDD card no. 044-0617), labeled (*) in Figure 2a, can be observed. The GO pattern shows the structure of a nano-graphene oxide (ICDD card no. 065-1528). The XRD patterns of all HT3Ce-xGO nanocomposites (Figure 2b) expose the same mixture of characteristic reflections of LDH and cubic cerianite CeO_2_ phases, with no additional impurity lines. The typical strong (001) basal diffraction peak of GO was not observed in all the nanocomposites. This could be attributed to the superimposed reflection with the (003) basal LDH peak and is also indicative of an exfoliation of the graphene sheets [46]. Apparently, the restacking of graphene sheets was inhibited by deposition of the LDH crystallites on the graphene sheets [47,48]. The structural data, the lattice parameters of the two phases, and their crystallite sizes are provided in Table 3. For the LDH phase, the crystallite sizes were calculated along two directions: perpendicular (D_003_) and parallel (D_110_) to the brucite-like layers, respectively. The data gathered in Table 3 show similar structural characteristics for all the samples. The CeO_2_ phase is structurally identical in all the samples, while the LDH-phase is more likely affected by the electrostatic interactions between GO and LDH during the synthesis [45,49]. As it can be observed, the crystallite sizes, in particular the coherence lengths in the layer-stacking direction (D_003_), were affected by the presence of GO. Indeed, the incorporation of GO led to the formation of larger LDH particles suggesting the involvement of the GO sheets as nucleating agents for the LDH phase formation [50]. The evolution in the HT3Ce-xGO series of the absolute intensities of the (110) line, exclusively related to the brucite-like layer, should go along with the decrease of the proportion of the LDH phase in the nanocomposites. This is the case, except for the inversion between the HT3Ce-20GO and HT3Ce-25GO samples, in line with the result obtained through textural measurements. Indeed, there is an obvious inverse correlation between the surface area and the I_110_(HT3Ce-xGO)/I_110_(HT3Ce-5GO) ratio (Figure S3), evidencing HT3Ce-5GO as the most “ordered” material, and HT3Ce-20GO as the most disordered one in this series. This order-disorder could be associated to the dispersion of HT3Ce on the GO sheets. HT3Ce-25GO appears to be more “ordered”, i.e., less dispersed, than HT3Ce-20GO likely due to GO restacking. 

A decrease of the *c*-lattice parameter value accompanied by an increase of the I_003_/I_110_ ratio for all the HT3Ce-xGO nanocomposites in comparison with the HT3Ce sample is observed (Table 3). The result suggests a slight modification of the interlayer anionic composition, probably a higher degree of hydration with a different compaction of the anionic species, due to mutual electrostatic interaction between LDH and GO phases. These observations are consistent with the DRIFT data presented afterward.

**Figure 3:**
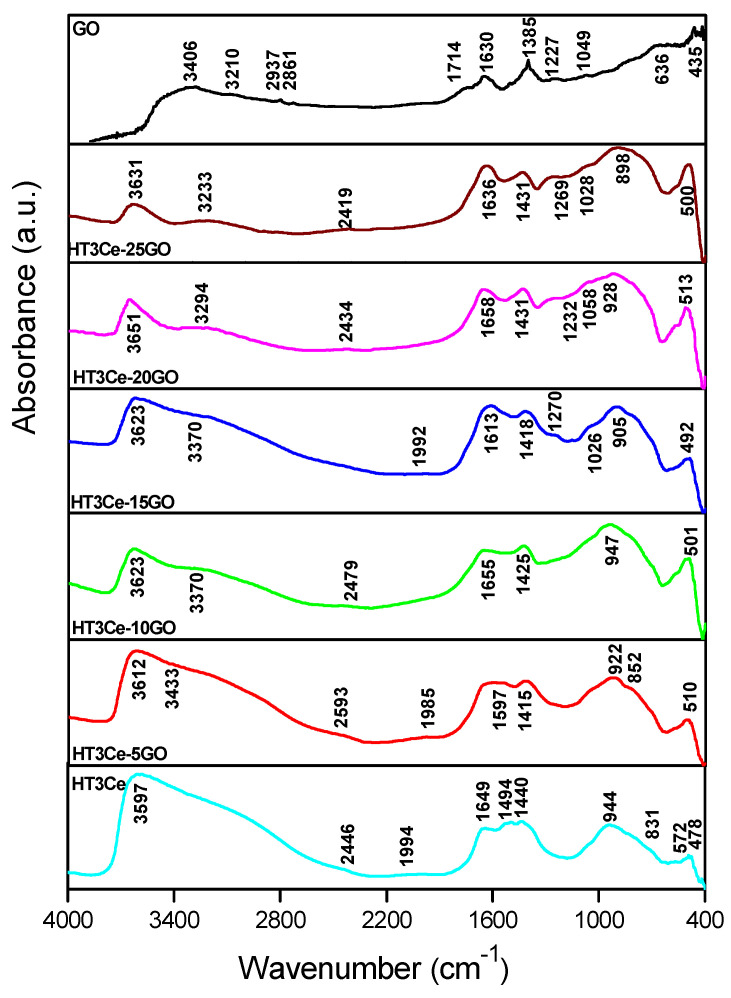
DRIFT spectra of the solids: HT3Ce-xGO composites, neat HT3Ce, and GO.

The DRIFT spectra of all the synthesized solids can be seen in Figure 3. The characteristics of the composites modified with GO were close to the HT3Ce structure, depicting the main layered phase. As the GO concentration increased in the hybrid solids, the stretching vibrations bands attributed to the OH groups present in the hydroxide layer of HT3Ce at 3587 cm^−1^ shifted towards larger values, namely 3612, 3623, 3623, 3651, 3631 cm^−1^, and their intensity also decreased. 

The same proportionality related to the GO concentration was observed influencing the shoulder displayed in the region at 3000–3200 cm^−1^ (probably due to the formation of hydrogen bonds between the water molecules). Furthermore, at 1645 cm^−1^, the absorption of the hydroxyl groups of the water molecules in the HT3Ce interlayer space was perturbed in the GO-containing composites and shifted towards 1597 (for HT3Ce-5GO), 1655 (for HT3Ce-10GO), 1613 (also wider, for HT3Ce-15GO), 1658 (for HT3Ce-20GO), and 1636 cm^−1^ (for HT3Ce-25GO). This aspect can be explained by the different confinement of the LDH particles on the larger GO layers, in good correlation with the already observed perturbation by the “turbostratic” effect [51]. 

The perturbation of the interlayer region appeared due to the favorable interactions between LDH and GO, mediated by ceria ions, which could be observed further. The asymmetric stretching vibrations of CO_3_^2−^ from HT3Ce were found at 1430 cm^−1^. The same peak (in the composites containing 5, 10, 15 wt.% GO) was shifted towards a lower absolute value (1415, 1425, 1418 cm^−1^), indicating a weaker interaction when GO was used. Moreover, the other CO_3_^2−^ vibration modes were affected in the same manner (in the 1495–1528 cm^−1^ region). 

At the same time, all the metal-oxygen vibrations in the 600–900 cm^−1^ region were shifted towards lower values indicating weaker energy states for the bending modes when GO was used. This aspect should also be viewed concerning the interlayer and edge interactions between LDH and GO. Other evidence on the perturbation of the interlayer region and the decrease in size of LDH elementary particles (as observed in the SEM section) were found in the case of HT3Ce-15GO, for which new absorptions were detected at 1270 and 1026 cm^−1^ (bands for the edge OH groups found in GO layers), also in HT3Ce-20GO (1232 cm^−1^, 1058 cm^−1^) and HT3Ce-25GO (1269 cm^−1^, 1028 cm^−1^).

**Figure 4:**
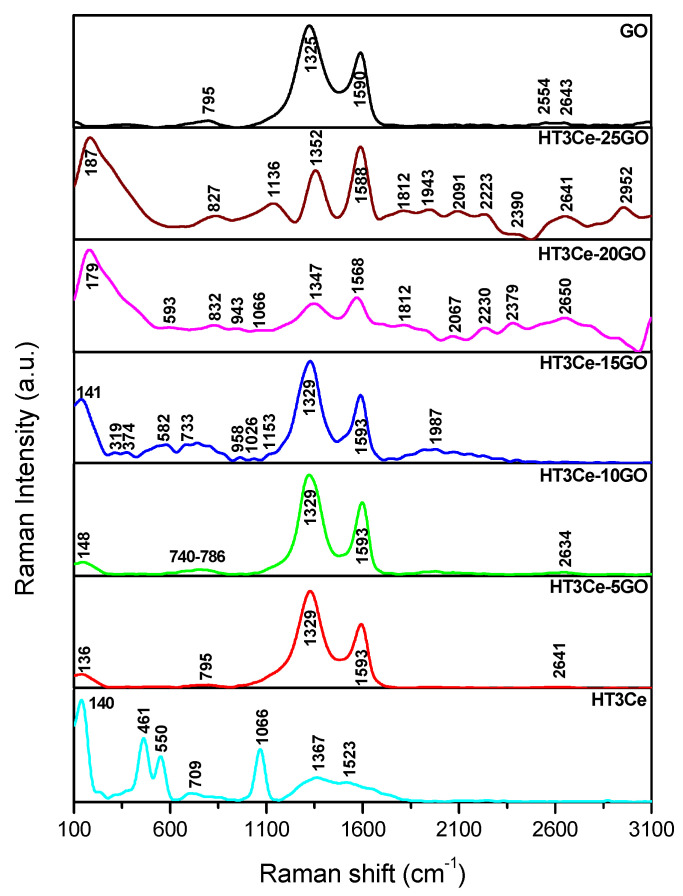
Raman spectra of the solids: HT3Ce-xGO composites, neat HT3Ce, and GO.

The presence of GO in all the HT3Ce-xGO composites was clearly evidenced by Raman spectra of the samples which are displayed in Figure 4. The spectrum of HT3Ce presented different bands, some specific to the LDH structure, namely a broad band characteristic to the lattice vibrations of the hydrotalcites at 140 cm^−1^, another one at 550 cm^−1^ attributed to stretching vibrations of the hydrogen bonds formed between the interlayer water and carbonate anions, and a band assigned to the symmetric stretching of the carbonate anion found at 1066 cm^−1^ [52]. In addition, a band was observed at 461 cm^−1^, characteristic to nanocrystalline CeO_2_ and some bands between 800 and 900 cm^−1^ specific for the stretching vibrations of peroxide groups and defective ceria surfaces [53].

The Raman spectrum of GO showed the D band at 1326 cm^−1^ and the G band at 1590 cm^−1^, while the ratio between the intensities of the two bands, which is associated to the surface defect and the degree of lattice distortion of a graphite layer within the carbon material [54], was I_D_/I_G_ = 1.38. Aside from the band at 137 cm^−1^ specific to LDH lattice vibrations in the Raman spectra of the HT3Ce-xGO composites, the GO component overlapped the bands characteristic for the LDH component. In the spectra of all the composites, the positions of the GO bands D and G were slightly shifted to higher values, precisely to 1329 and 1593 cm^−1^ for the samples containing 5, 10, and 15 wt.% GO, and 1347 and 1590 cm^−1^ for the samples containing 20 and 25 wt.% GO, while the I_D_/I_G_ ratio increased with the GO content in the sample to 1.5, 1.43, and 1.53 for 5, 10, and 15 wt.%, respectively, indicating an increased disturbance of the GO layer. The situation changed for the samples containing 20 and 25 wt.% GO, the I_D_/I_G_ ratio decreased with the GO content in the sample to 0.89 and 0.92 cm^−1^, respectively. This could be due to GO restacking at high GO contents, as also suggested by the evolution of the surface area of the samples. Another interesting aspect noticed in the Raman spectra was the presence of the G’ band, which appeared at around 2600 cm^−1^. This band increases with the concentration of graphene oxide inserted in the composites. Apparently, with the increase of the GO concentration, the hydrotalcite crystallized on the surface of the graphene oxide, an observation also supported by the SEM images.

**Figure 5:**
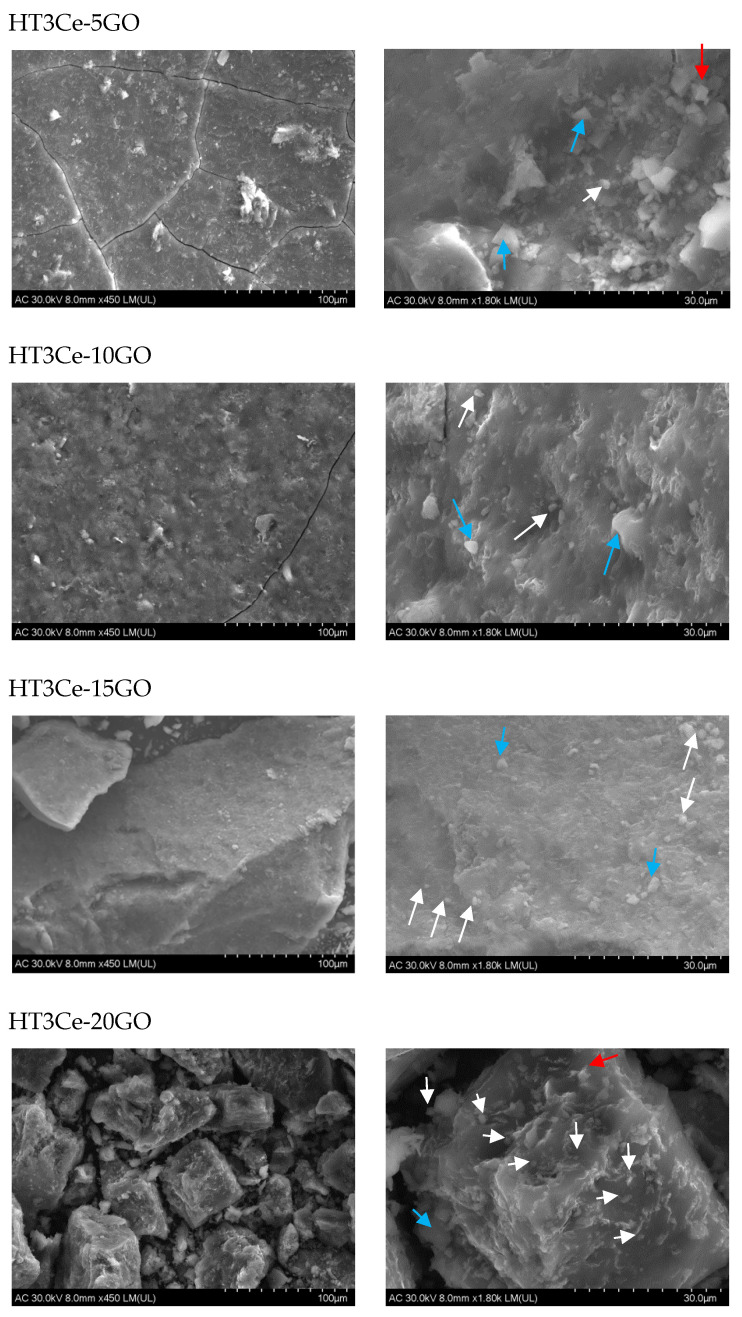

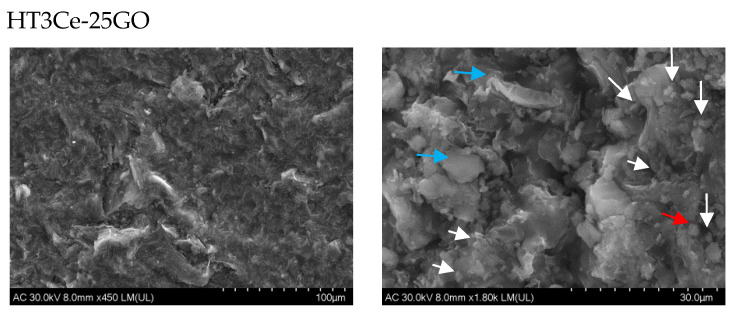
SEM images of the HT3Ce-xGO composites (left: low magnification; right: high magnification), with different aspects of the grain surfaces indicating polymorphic (blue arrows), irregular “ovoidal” (white arrows), or cubic (red arrows) structures.

The composite structures, analyzed in powder form by SEM, highlighted different surface morphologies of the HT3Ce-xGO samples (Figure 5). A closer inspection of the grain surface revealed the presence of microparticles grouped either in polymorphic structures with sharp edges, “ovoidal” particles, or cubic agglomerates (Figure 5). It is noteworthy that the morphology of the neat Ce-containing MgAl LDH sample was shown to be quite different [55], being described as assemblies of nanoplates nearly perpendicular to the outer surfaces of microspheres.

The number (frequency) of “ovoidal” particles on the grain surface increased with GO concentration, but their dimension decreased. This fact also determined a slight twist of the surface layers beyond the 15% GO concentration.

A possible explanation of the overall mechanism for the occurrence of the “ovoidal” composite particles and their positioning on the surface could also be related to the complex role played by Ce ions. On one hand, they can influence the oxidative-reductive equilibrium and act on (not fully oxidized) GO sites, and, on the other hand, they could induce a basic character in the LDH reaction sites, leading to a spherical association of the hexagonal LDH platelets [56]. 

The “sensitivity” of the isoelectric point (IEP) for LDH during synthesis was broadly described in the literature [56,57]. Over the IEP, a spherical association of hexagonal LDH platelets occurs. The formation of “ovoidal” composite particles was possible, in a larger context, due to pH conditions and GO availability for offering a large surface area (useful for both LDH and ceria particles). Moreover, it was reported that the ceria phase could be attached to the GO surface preventing the formation of extrinsic vacancies in the oxygen sub-lattice [53]. By this mechanism, a larger specific area with a higher number of active centers could be obtained for the catalytic reactions [58]. 

### 3.2. Catalytic Tests

First, the Knoevenagel condensation of benzaldehyde with diethyl malonate catalyzed by various Mg_3_Al_0.75_Ce_0.25_ LDH-GO composites containing different concentrations of GO in the range of 5–25 wt.% resulted in diethyl benzylidene malonate (DBM). It was immediately converted to a certain extent in the presence of the basic catalyst according to a Doebner-like modification of the Knoevenagel reaction to cinnamyl ethyl ester (ECE), which was finally cleaved to cinnamic acid (CA) as a second main reaction product (Scheme 1). The values of conversions and selectivities after 5 and 24 h reaction time, respectively, are presented in Table 4. 

As it can be observed in Table 4, the catalytic activity in the Knoevenagel condensation passes through a maximum for the system containing 15% GO. This may be due to the fact that HT3Ce-15GO is the most basic system in the HT3Ce-xGO series, with the highest basic/acidic sites ratio (Table 1). At the same time, for neat HT3Ce and GO alone, the conversions obtained were rather small compared to the hybrid composites. This suggests a synergistic effect between the parent materials (GO and LDH) present in the HT3Ce-xGO composites leading to improved catalytic activity. The higher activities of the hybrid HT3Ce-xGO systems compared to the neat HT3Ce catalyst could be due to their strong affinity for the organic substrate since graphene oxide presents a similar planar structure with the phenyl group. Moreover, the association of composite platelets in “ovoidal” particles in the hybrid materials, as evidenced in the SEM images (Figure 5), could also be correlated with their enhanced activity compared to neat HT3Ce that shows no evident “ovoidal” particles [55]. 

Notably, a longer reaction time led to higher conversions of the substrate, with a conversion increase larger for the hybrid HT3Ce-xGO systems compared to pure HT3Ce and GO, in line with the higher activity of the former. 

**Figure 6:**
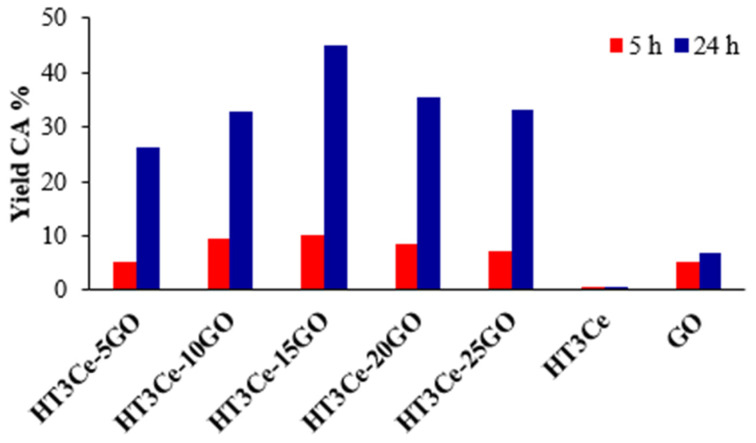
The yields of CA obtained with the investigated catalysts, namely HT3Ce-xGO (*x* = 5, 10, 15, 20, 25 wt. % GO), HT3Ce, and GO.

The yields of CA, a product of high interest, obtained with the investigated catalysts are illustrated in Figure 6. It can be observed that the yield passes through a maximum for the HT3Ce-15GO catalyst. 

**Figure 7:**
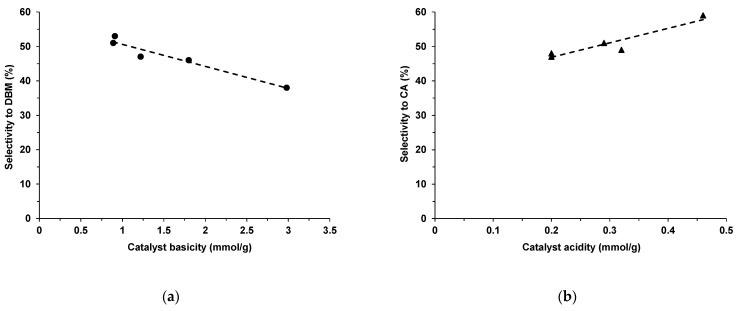
Selectivity to DBM after 24 h reaction time vs. catalyst basicity (**a**) and selectivity to CA after 24 h reaction time vs. catalyst acidity (**b**) for the HT3Ce-xGO composites in the Knoevenagel condensation of benzaldehyde with diethylmalonate.

The basicity of the catalyst should determine not only the activity but also the selectivity to diethyl benzylidene malonate (DBM), as the basic sites are involved in the proton abstraction from the α-position of diethyl malonate then the resulting anion undergoes nucleophilic addition to the carbonyl group of the benzaldehyde, thus yielding to diethyl benzylidene malonate. However, surprisingly, the selectivity to DBM decreases with increasing the basicity of the catalyst (Figure 7a). This could be explained by the fact that the decarbethoxylation of DBM in the solvent phase, first leading to the ethyl cinnamate ester intermediate, is also favored by the basicity of the catalyst. At the same time, the conversion of the latter into cinnamic acid (CA) seems to be favored by the acid sites of the catalytic material, as suggested by the linear correlation observed between the selectivity to CA and the acidity of the catalyst (Figure 7b). Moreover, judging by the high selectivities for CA of pure GO phase (Table 4), the latter plays a key role in the cinnamic acid production obviously through the acid sites created in the hybrid materials. These data suggest that the ratio between the acid and basic sites in the hybrids is a key factor determining the product distribution. However, the fact that solids like HT3Ce-10GO and HT3Ce-25GO, which have quite different basic-to-acidic sites ratios (Table 1) show similar selectivities to CA for the reaction after 24 h, although quite different after only 5 h (Table 4), suggests that the strength of the acidic and basic sites also plays an important role. 

**Figure 8:**
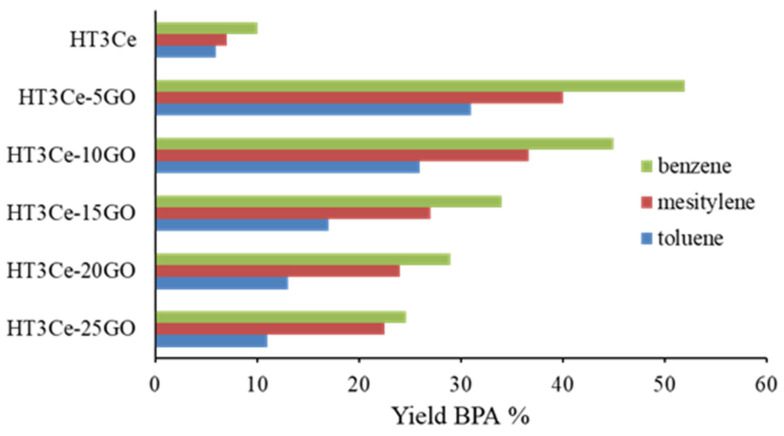
The yields of 2-benzoyl-3-phenylacrylonitrile (BPA) obtained at the end of the cascade reaction for each cerium-containing LDH-GO composite catalyst.

The conversion values for the one-pot oxidation-Knoevenagel condensation cascade reaction and the yields to 2-benzoyl-3-phenylacrylonitrile (BPA) can be seen in Table 5 and Figure 8, respectively. 

HT3Ce-xGO composites exhibited a bifunctional catalytic behavior and could promote aromatic alcohol oxidation/Knoevenagel condensation in a stepwise way. From the already existing information in the open literature [59], low yields of oxidation products were obtained when polar solvents, such as acetonitrile, methanol, and ethanol, were utilized. Therefore, three different non-polar solvents were used for the current reactions, namely toluene, benzene and mesitylene. 

According to the data presented in Table 5, the best conversion values were obtained when benzene was used as a solvent. Although it is a hazardous and carcinogenic compound, benzene is still a solvent appropriate for manifold reactions because it is inert and stable since its π electrons are maximally spread out evenly. Mesitylene and toluene were also suitable solvents, but their use led to the formation of a larger number of byproducts.

Irrespective of the solvent used, the conversion of benzyl alcohol increased with the decrease of the graphene oxide concentration in the hybrid composite. This is not surprising if one takes into consideration that from the two pure materials, HT3Ce and GO, only the LDH is active, the GO being completely inactive. However, a synergistic effect between the two phases in the hybrid HT3Ce-xGO materials can be noticed, which is diminished, in this case, by a dilution effect with increasing the GO content. Taking into consideration that both redox (associated to cerium species) and basic active sites involved in the studied cascade reaction are contained by the LDH phase, the addition of GO to obtain the composites likely increases the accessibility to the catalytic sites by separating the LDH particles. Further increase of the GO content results in a decrease of the number of redox active sites involved in the first step of the cascade reaction, in line with the observed decrease of Ce content (Table 1) with negative consequences on the overall activity. Thus, practically, HT3Ce-5GO catalyst gave the highest conversion of benzyl alcohol in the series studied. Furthermore, the highest yield of 2-benzoyl-3-phenylacrylonitrile was achieved at the end of the cascade reaction with this catalytic system, as seen in Figure 8. Nevertheless, it is noteworthy that the conversion of benzyl alcohol is significantly lower for HT3Ce-25GO catalyst compared to HT3Ce-20GO, although they have similar Ce content. This could be attributed to the lower surface area of the former associated to a diminished dispersion of HT3Ce due to the GO restacking at higher GO content in the hybrid, which led to a limited accessibility to the redox sites.

## 4. Conclusions

The co-precipitation of Ce-containing MgAl-LDH in the presence of a GO suspension led to HT3Ce-xGO composites with increased crystallinity as indicated by the XRD analysis. The diffractograms of the composites also showed that no other impurities could be found besides the CeO_2_ phase. Raman spectroscopy clearly highlighted the existence of GO in the HT3Ce-xGO composites, while the SEM images demonstrated that the elementary particles were grouped either in layered polymorphic particles with edges, “ovoidal” composite particles, or cubic agglomerates, and the amount of ovoidal particles increased with GO content.

The catalytic activity of the obtained LDH-GO composites was tested in two reactions, namely the Knoevenagel condensation between benzaldehyde and diethyl malonate and the tandem aerobic oxidation of benzyl alcohol followed by Knoevenagel condensation with benzoyl acetonitrile.

The following observations were established for the Knoevenagel condensation reaction:

- Using the unmodified solids HT3Ce and GO, the conversions obtained were rather low compared to the hybrid composites. A synergistic effect between the LDH and GO parent materials present in the HT3Ce-xGO composites, leading to improved catalytic activity, was noticed.
- The catalytic activity of the hybrids increased with the GO content in the composite catalysts up to an optimum for HT3Ce-15GO system, then it decreased for higher GO content. This follows the evolution of the number of basic sites in the catalytic material.
- The ratio between the basic and acid sites in the hybrids, associated to the LDH and GO phases, respectively, is a key factor determining the product distribution.
- A longer reaction time led to higher conversions of the substrate, with a conversion increase larger for the hybrid HT3Ce-xGO systems compared to their pure constituents.

In the case of the tandem reaction, the conversion of benzyl alcohol was higher for the hybrid HT3Ce-xGO systems compared to the LDH and GO materials alone, the latter being completely inactive, but it decreased with the increase of the GO concentration. This behavior was attributed to the fact that the addition of GO to LDH to obtain the composites increases the accessibility to the redox sites needed in the first sequence of the tandem reaction located in the LDH material by separating its particles. Then, further increasing the GO content results in a decrease of the number of redox active sites and, thus, of both conversion and product yield. At high GO content, the GO restacking takes place, the accessibility to the catalytic sites being unfavorably affected. Notably, the conversion of benzyl alcohol depended on the solvent used, the most suitable solvent being benzene.

## Figures and Tables

**Table 1:** Chemical composition determined by XRF and the results of acidity-basicity measurements.

Catalyst	Chemical Composition					Acidity and Basicity Measurements			
	Mg^2+^ (wt.%)	Al^3+^ (wt.%)	Ce^3+^ (wt.%)	Mg/(Al+Ce) Atomic Ratio	Ce/Al Atomic Ratio	Total Acidic Sites (mmol Pyridine/g)	% HB ^1^	Total Basic Sites (mmol Acrylic Acid/g)	Basic/Acidic Sites Ratio
HT3Ce	17.53	5.71	8.91	2.65	0.30	0.03	63.4	0.78	26.00
HT3Ce-5GO	16.31	5.10	8.30	2.74	0.31	0.20	6.7	0.91	4.55
HT3Ce-10GO	15.34	5.05	7.88	2.63	0.30	0.32	18.8	0.89	2.78
HT3Ce-15GO	11.75	3.72	5.72	2.74	0.30	0.46	20.0	2.98	6.48
HT3Ce-20GO	10.89	3.24	4.95	2.92	0.29	0.29	25.4	1.80	6.21
HT3Ce-25GO	10.21	3.23	5.00	2.74	0.30	0.20	43.2	1.22	6.10
GO	-	-	-	-	-	0.77	31.2	0.06	0.08

^1^ Brønsted acid sites.

**Table 2:** Textural properties of the synthesized samples.

Samples	Surface Area (m^2^/g)	Pore Volume (cm^3^/g)	Pore Size ^a^ (Å)
HT3Ce	15.0	0.063	126.1
HT3Ce-5GO	35.5	0.079	185.6
HT3Ce-10GO	42.0	0.089	37.5 and 75.9
HT3Ce-15GO	51.7	0.117	37.8 and 92.8
HT3Ce-20GO	77.6	0.144	34.7 and 50.8
HT3Ce-25GO	68.3	0.142	35.5 and 72.1
GO	79.8	0.071	38.9

^a^ Maxima of pore size distribution.

**Table 3:** Crystal unit cell parameters of the synthesized samples.

Samples	LDH					CeO_2_	
	*a* (Å)	*c* (Å)	I_003_/I_110_	D_110_ (nm)	D_003_ (nm)	*a* (Å)	D_111_ (nm)
HT3Ce-5GO	3.068	23.658	5.07	12.9	8.4	5.394	3.2
HT3Ce-10GO	3.067	23.660	5.41	15.4	8.2	5.395	3.2
HT3Ce-15GO	3.066	23.629	5.62	15.1	8.8	5.398	3.2
HT3Ce-20GO	3.068	23.681	5.96	12.0	7.3	5.405	3.3
HT3Ce-25GO	3.065	23.572	5.65	14.0	7.7	5.401	3.2
HT3Ce	3.066	23.756	4.85	11.7	6.3	5.415	4.6

**Table 4:** Catalytic results of the HT3Ce-xGO composites containing five different concentrations of GO (*x* = 5, 10, 15, 20, 25 wt. % GO) at 5 and 24 h reaction time.

Catalyst	Conversion of Aldehyde (%)		Selectivity (%) After 5 h			Selectivity (%) After 24 h		
	5 h	24 h	DBM	ECE	CA	DBM	ECE	CA
HT3Ce-5GO	14	56	54	7	39	53	-	47
HT3Ce-10GO	19	67	51	-	49	51	-	49
HT3Ce-15GO	24	76	56	-	44	38	3	59
HT3Ce-20GO	21	70	59	-	41	46	3	51
HT3Ce-25GO	18	69	62	-	38	47	5	48
HT3Ce	8	9	57	37	6	58	34	8
GO	5	7	-	1	99	-	5	95

**Table 5:** Catalytic results of the HT3Ce-xGO composites containing different concentrations of GO (*x* = 5, 10, 15, 20, 25 wt.% GO) in the one-pot oxidation–Knoevenagel condensation cascade reaction performed in different solvents: toluene, mesitylene, and benzene.

Catalyst	Conversion of Benzyl Alcohol (%) in The Following Solvents:		
	Toluene	Mesitylene	Benzene
HT3Ce-25GO	14	17	38
HT3Ce-20GO	24	38	45
HT3Ce-15GO	25	38	49
HT3Ce-10GO	40	42	52
HT3Ce-5GO	40	45	53
HT3Ce	11	22	30
GO	0	0	0

## Data Availability

The data presented in this study are available on request from the corresponding authors.

## Supplementary Materials

The following are available online at https://www.mdpi.com/article/10.3390/ma14237457/s1, Figure S1: Evolution of the cationic content vs. GO content in the HT3Ce-xGO hybrid samples, Figure S2: Pore size distributions of the GO, HT3Ce, and HT3Ce-xGO samples, Figure S3: Evolution of the I110(HT3Ce-xGO)/I110(HT3Ce-5GO) ratio vs. surface area in the HT3Ce-xGO series.
