# Supplementary material for: Ce-Containing MgAl-Layered Double Hydroxide-Graphene Oxide Hybrid Materials as Multifunctional Catalysts for Organic Transformations
Source: Materials (Basel). 2021 Dec 4;14(23):7457. doi: 10.3390/ma14237457 (PMC8659285; doi:10.3390/ma14237457)
Supplement: Supplementary file 1 [file materials-14-07457-s001.zip › materials-1456337-supplementary.pdf]

materials

MDPI

Supplementary Materials

Ce-Containing MgAl-Layered Double Hydroxide-Graphene Oxide Hybrid Materials as Multifunctional Catalysts for Organic Transformations

Alexandra-Elisabeta Stamate [1,2], Octavian Dumitru Pavel [1,2], Rodica Zăvoianu [1,2,*], Ioana Brezeştean [3,4], Alexandra Ciorîță [3,5], Ruxandra Bîrjega [6], Katja Neubauer [7], Angela Koeckritz [7] and Ioan-Cezar Marcu [1,2,*]

[1] Department of Organic Chemistry, Biochemistry & Catalysis, Faculty of Chemistry, University of Bucharest, 4-12, Blv. Regina Elisabeta, 030018 Bucharest, Romania; alexandra-elisabeta.stamate@drd.unibuc.ro (A.-E.S.); octavian.pavel@chimie.unibuc.ro (O.D.P.)
[2] Research Center for Catalysts & Catalytic Processes, Faculty of Chemistry, University of Bucharest, 4-12, Blv. Regina Elisabeta, 030018 Bucharest, Romania
[3] National Institute for Research and Development of Isotopic and Molecular Technologies, 67-103 Donat, 400293 Cluj-Napoca, Romania; ioana.brezestean@itim-cj.ro (I.B.); al.ciorita@yahoo.com (A.C.)
[4] Biomolecular Physics Department, Faculty of Physics, Babes-Bolyai University, 1, Kogălniceanu Str., 400084 Cluj-Napoca, Romania
[5] Electron Microscopy Centre, Faculty of Biology and Geology, Babes-Bolyai University, 44, Republicii Str., 400015 Cluj-Napoca, Romania
[6] National Institute for Lasers, Plasma and Radiation Physics, 409 Atomistilor Street, P.O. Box MG-16, 077125 Măgurele, Romania; ruxandra.birjega@inflpr.ro
[7] Leibniz Institute for Catalysis (LIKAT Rostock), Albert-Einstein-Straße 29a, 18059 Rostock, Germany; katja.neubauer@catalysis.de (K.N.); angela.koeckritz@catalysis.de (A.K.)
* Correspondence: rodica.zavoianu@chimie.unibuc.ro (R.Z.); ioancezar.marcu@chimie.unibuc.ro (I.-C.M.)

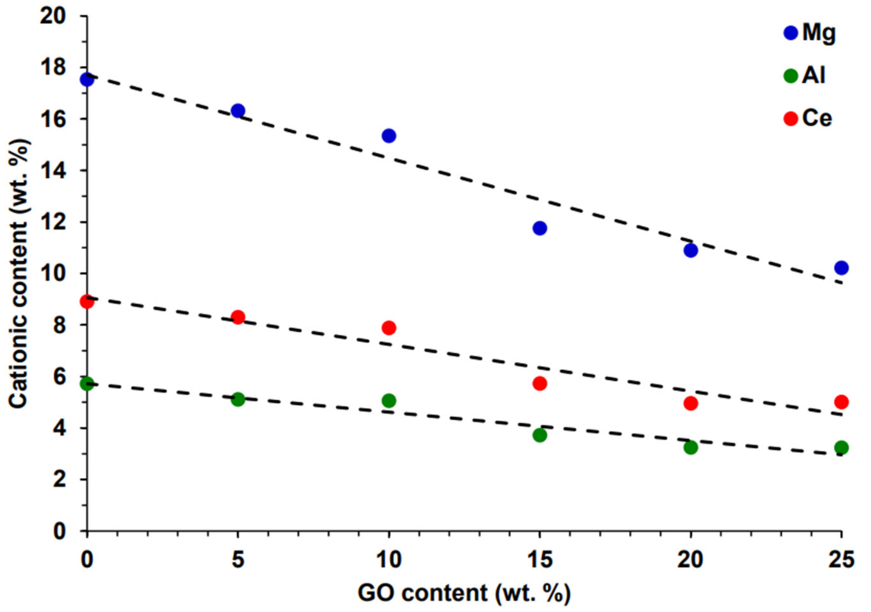

Figure S1. Evolution of the cationic content vs. GO content in the HT3Ce-*x*GO hybrid samples.

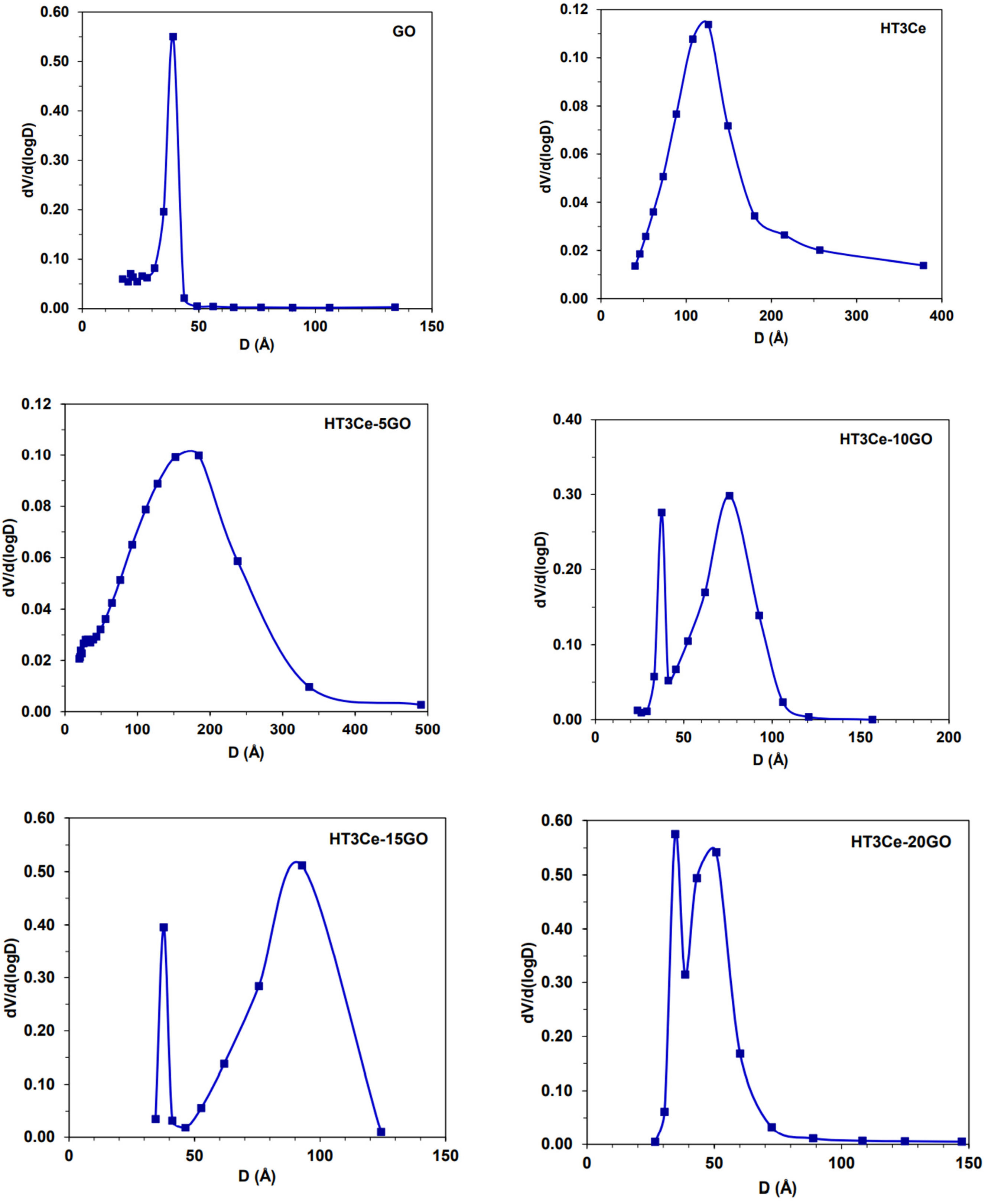
GO
HT3Ce
HT3Ce-5GO
HT3Ce-10GO
HT3Ce-15GO
HT3Ce-20GO
dV/d(logD)
D (Å)

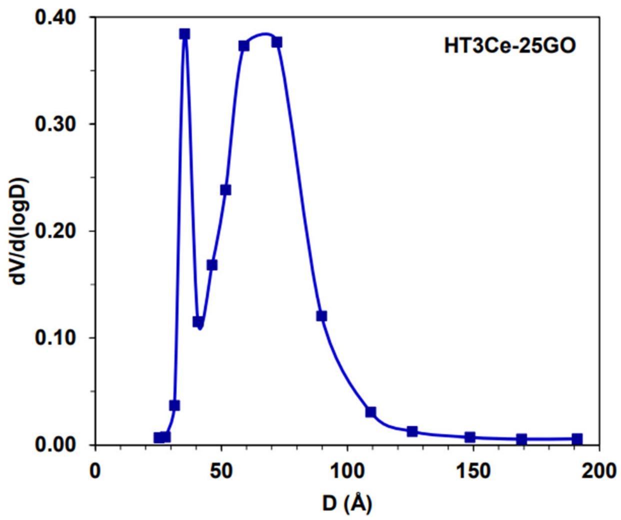

Figure S2. Pore size distributions of the GO, HT3Ce and HT3Ce-*x*GO samples.

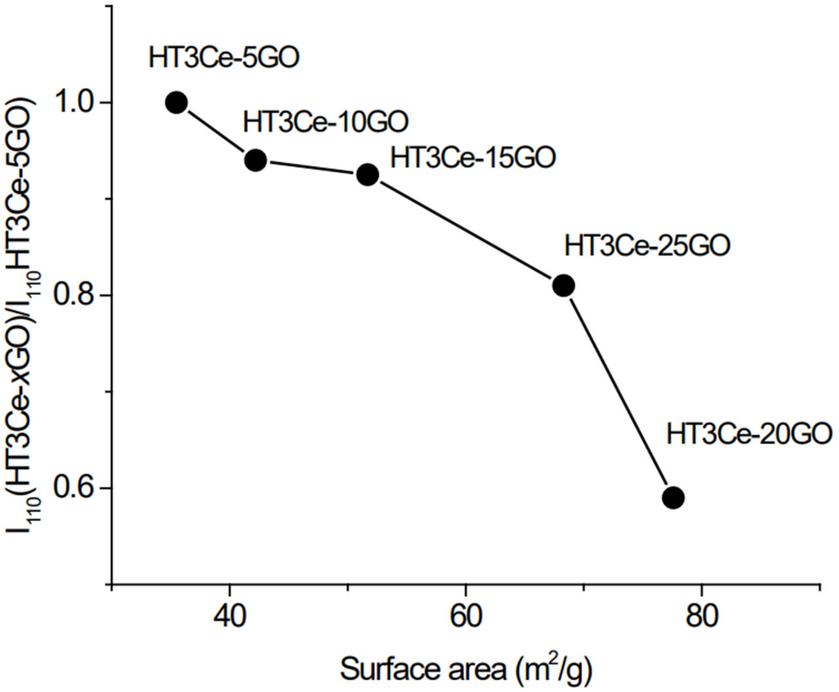

Figure S3. Evolution of the I_{110}(HT3Ce-*x*GO)/I_{110}(HT3Ce-5GO) ratio vs. surface area in the HT3Ce-*x*GO series.
